# Eye tracking in catheter-based cardiovascular interventions: early results

**DOI:** 10.1117/1.JMI.4.3.035502

**Published:** 2017-08-04

**Authors:** Peter Lanzer, Mohammad Al-Naser, Syed Saqib Bukhari, Andreas Dengel, Elizabeth A. Krupinski

**Affiliations:** a Middle German Heart Center and Division of Cardiovascular Disease, Center of Internal Medicine, Health Care Center Bitterfeld-Wolfen gGmbH, Bitterfeld-Wolfen, Germany; b University of Kaiserslautern, Department of Computer Science, Kaiserslautern, Germany; c German Research Center for Artificial Intelligence, Smart Data & Knowledge Services Department, Kaiserslautern, Germany; d Emory University, Department of Radiology and Imaging Sciences, Atlanta, Georgia, United States

**Keywords:** catheter-based cardiovascular interventions, teaching, perception, eye tracking

## Abstract

Visual x-ray image processing (XRIP) represents a fundamental component of catheter-based cardiovascular interventions (CBCVIs). To date, no data are available to define XRIP in this setting. To characterize CBCVI XRIP, we developed a computer-based method allowing continuous temporal–spatial analysis of data recorded by a head-mounted eye-tracking device. Quantitative analysis of gaze duration of an expert operator (EO) revealed that the average time in minutes spent viewing the images on the display screen was 39.5%±13.6% and 41.5%±18.3% of the total recorded time in coronary angiography (CA) and in CA followed by CBCVI, respectively. Qualitative analysis of gaze data of the EO revealed consistent focus on the center point of the screen. Only if suspicious findings were detected did gaze move toward the target. In contrast, a novice operator (NO) observing a subset of cases viewed coronary artery segments separately and sequentially. The developed methodology allows continuous registration and analysis of gaze data for analysis of XRIP strategies of EOs in live-cases scenarios and may assist in the transfer of experts’ reading skills to novices.

## 1 Introduction

Catheter-based cardiovascular interventions (CBCVIs) are primarily guided by the human observer’s rapid x-ray image processing (XRIP) and interpretation processes. While x-ray image quality is steadily improving due to better image acquisition^1^ and postprocessing techniques,^2^ the principal limitations, the two dimensional projectional character and inability to visualize arterial walls, remain.^3^^,^^4^ Image-based guidance in CBCVI requires optimum image acquisition (e.g., appropriate angulation, zooming, and shielding), as well as optimum image interpretation. The latter is based on reading skills of the operators and their ability to identify and interpret pathological findings rapidly, reliably, and efficiently, particularly in emergency settings.^5^ To date, CBCVI image reading skills are largely acquired empirically. Consequently, their nature remains largely obscure. We performed a pilot study to validate the use of eye-tracking technology to allow analysis and comparison of reading strategies of operators reading films during real-life interventions. We hypothesized that optimum image viewing skills are based on similar principles, allowing CBCVI operators to read-out large amounts of image data in real time and on-line. Characterization, externalization, and transfer of these fast and frugal strategies could reduce the length of learning curves and improve their reproducibility when compared with the current poorly defined and often incidental learning.

While, to our knowledge, studies on XRIP in CBCVI have not been performed to date, a related study investigating the ability of radiology residents to make correct diagnosis using simulation technology was previously performed.^6^ Similar studies have been conducted in other interventional procedure medical tasks,^7^^–^^10^ but again, to our knowledge, no studies have been performed in real-life settings to date. The aim of this pilot study was to develop and validate a methodology for safe and reliable detection and analysis of the gaze data of operators during live CBCVI to be employed in future studies.

## 2 Methods

### 2.1 Study Protocol

All procedures were performed in the catheterization laboratory of the Health Care Center (HCC) in Bitterfeld-Wolfen gGmbH between July 7, 2015, and November 11, 2015. In all patients, established standard operating protocols (SOP) pertaining to CBCVI in HCC were followed. As no deviations from the SOPs were allowed or necessary, no confounding variables other than those accompanying any real-life intervention were introduced. All x-ray images were displayed in standard grayscale format, so no tests for color vision were required. All procedures were performed by a single expert operator (EO). Fourteen patients, four females (mean age 69.3±11.1) and 10 males (mean age 67.3±8.7) underwent CBCVI, including six coronary angiographies (CAs), six single stage CAs and percutaneous coronary interventions (PCI), one single stage peripheral angiography (PA) and angioplasty, and one 4-vessel cerebral angiography (4VCA) and carotid artery stenting (CAS). Two of the PCIs were emergency procedures. Standard CA protocol included four projections of the left coronary artery (LCA) and three projections of the right coronary artery (RCA). These projections were left anterior oblique (LAO) with cranial tilt, right anterior oblique (RAO) with cranial tilt, RAO with caudal tilt and LAO with caudal tilt for the LCA and LAO, and LAO with cranial tilt and RAO for the RCA. Standard PA protocol included sequential overlapping digital subtraction angiographic (DSA) images of the pelvic arteries and arteries of both legs in posterior–anterior (PA) projections. Standard imaging protocol of the 4VCA included DSA images of the aortic arch, extra- and intracranial segments of both carotid and both vertebral arteries in PA, and PA with cranial tilt and lateral projections. In cases of suspicious findings identified by the EO, additional views to depict the suspicious regions were acquired. The study was approved by the ethical review board.

**Fig. 1:**
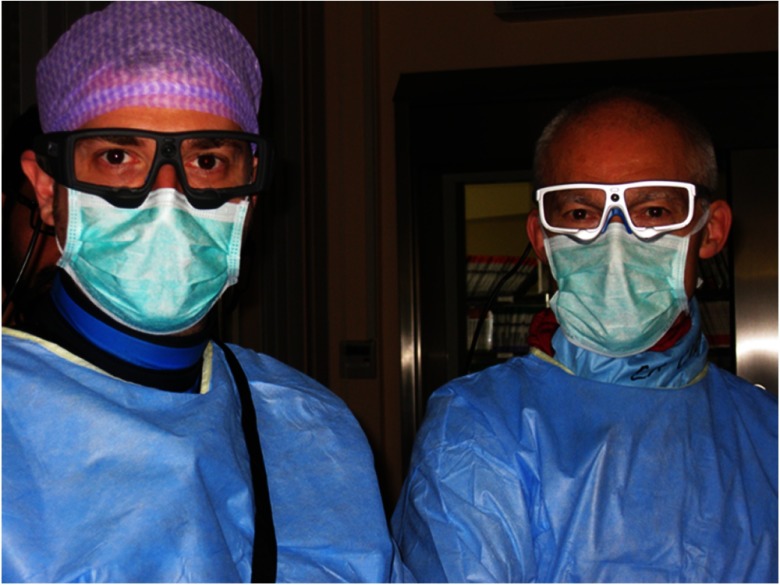
Operators with SMI ETG 2.7 eye trackers. Shown are the two operators participating in the study.

The eye-tracking data of the EO were recorded with SensoMotoric Instruments (SMI), Teltow, Germany, ETG 2.7 eye-tracking device. (SensoMotoroc Instruments, Teltow, Germany). This head-mounted eye tracker operates at 60-Hz frequency with reported accuracy from the manufacturer of 0.5 deg. The eye-tracking device has similar dimensions and appearance to eyewear routinely used by the operators for x-ray protection; therefore, the wearer’s vision and ability to view anything in the visual field corresponded to the real-life settings (Fig. 1). However, operators with impaired vision would need to use contact lenses rather than spectacles to accommodate the eye tracker. Recording started at the beginning of the procedure and terminated at the procedure’s end. Due to the lack of the availability of a second eye tracker, only in three cases did a novice operator (NO) (board-certified cardiologist and first-year fellow in interventional cardiology) follow the procedure wearing the eye-tracking device and standing next to the EO. To ensure that the EO and NO viewed the images independently, in contrast to the standard real-life scenario, no verbal exchange between the EO and NO was permitted. Case complexity was judged by the EO based on the presence and severity of tortuosities, calcifications, and morphological characteristics of the target lesions in PCI and based on the form of the aortic arch, elongation of the common carotid artery, and characteristics of the target lesions in CAS.

The recording device was placed into a pouch fastened on the back of the operators. The angulation of all projections and the length of imaging sequences were all decided by the EO. The visual angle was 16.26 deg.

To synchronize the eye tracker with the cine and fluoroscopy image data, a stationary camera was installed in the control room facing the control monitor displaying all images. Both eye tracker and stationary camera were started at the beginning (local anesthesia at the access site) and switched off at the end (final image acquisition) of procedures.

To map the gaze data, nine ArUco markers^11^ were attached to the frame of a life monitor with a resolution of 1200×1200  pixels per inch (ppi) located in the catheterization laboratory facing the EO. This monitor displayed all acquired fluoroscopic, cine, and DSA x-ray images simultaneously with the control monitor with a resolution of 1600×1200  ppi located outside the laboratory. A second reference monitor with a resolution of 1200×1200  ppi was positioned above the monitor in the catheterization laboratory to store images required to guide interventions. It was not used in any of the diagnostic angiographies. The monitor in the control room was not used for image viewing, but rather the video recording of this monitor was used to synchronize the data between the recording of the eye-tracking device and the course of the procedures. Markers established correspondence points between the display in catheterization laboratory and the display in the control room for gaze data mapping between the eye-tracker video and the video from the camera installed in the control room. The eye-tracking device was calibrated before the recording started by having the operator direct his gaze successively on each marker attached to the life monitor. In all cases, there was no interference of any of the eye tracking-related equipment with the standard conduct of the CBCVI procedures.

### 2.2 Data Analysis

**Fig. 2:**
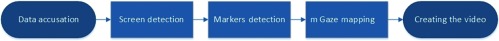
Video creation steps. The steps shown include both image acquisition and image processing.

The data from the eye tracker were mapped into the video recorded in the control room using the steps shown in Fig. 2.

**Fig. 3:**
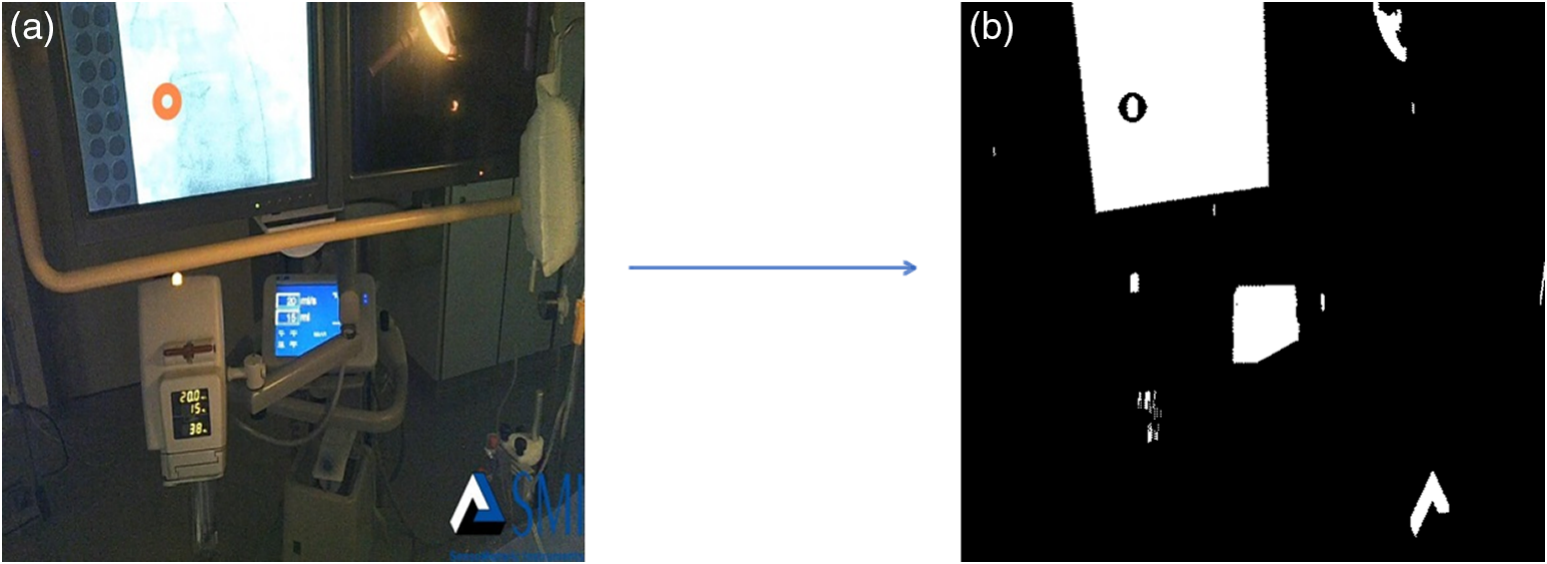
(a) The view of the monitor in catheterization laboratory and (b) corresponding frame after the thresholding and morphological process.

The first step was data acquisition. For the second step consisting of data processing, a shape-based^12^^–^^14^ image processing method was used. This technique is based on the fact that the screen is always the brightest and largest object in the frame, provided it was present in a given frame. Moreover, the screen has a square shape. Therefore, to find the frames with the screen, we used the blue color channel as it optimally differentiated bright and dark objects. Subsequently, the frames were thresholded^15^^,^^16^ to convert them into binary format. The threshold method converts pixels with intensity values more than a constant I to white pixels and pixels with intensity values less than the constant I to black pixels. Subsequently, we performed morphological operations to generate images as shown in Fig. 3.

These image region properties were then used to find the object with the largest area. To confirm that the area detected was indeed the monitor, we found the smallest bounding box contacting the identified object and then subtracted the area of the bounding box from the object itself. As a result, we found a significant difference between the screen and any other objects because the screen forms a square while other objects have random shapes.

**Fig. 4:**
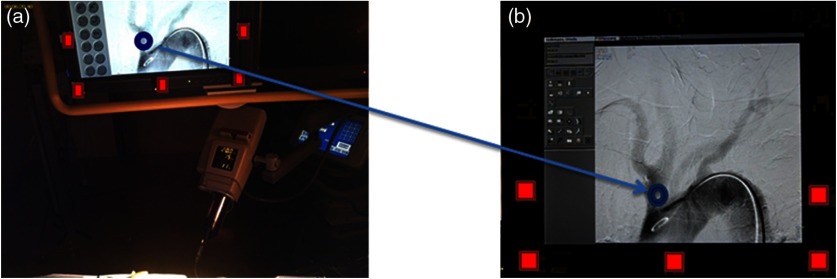
Gaze mapping using homography transformation method. (a) A frame from the eye tracker with five markers detected (colored with red) and (b) the correspondent frame from the stationary camera video. In both images, the blue circle represents the gaze.

A color-based detection method^15^ was used in the third step—marker detection. Since we detected the screen and found the bounding box that contains it, we used it to detect the markers distributed around the screen. Our goal was to find the lower five markers (Fig. 4), since the screen in many frames was not fully captured by the camera. To detect the markers, the corners of the bounding box were located, and the lower two corners were used to locate the markers based on their intensity since they were the only white objects around the screen.^12^^–^^14^

To map the gaze in step four, the homography transformation^17^ was used to transform the data from the video recorded in the control room. To create the transformation matrix, five points from the eye-tracker video and their corresponding points (lower five markers’ centers) from the control room video were used. Since the camera was fixed during recording, we manually selected the mutual points and used them for all the frames. Then, we used the following equation to map the gaze:

$$[\begin{array}{c} x^{\prime }\\ y^{\prime }\\ 1 \end{array}]=[\begin{array}{ccc} h_{11} & h_{12} & h_{13}\\ h_{21} & h_{22} & h_{23}\\ h_{31} & h_{32} & h_{33} \end{array}]\times [\begin{array}{c} x\\ y\\ 1 \end{array}],$$

where x and y are the input gaze coordinates from the eye tracker, $h_{ij}$ is the transformation matrix created by the homography method, and $x^{\prime },y^{\prime }$ are the gaze coordinates mapped to the high-quality video. We then calculated the homography transformation matrix for each frame. The resulting gaze points were mapped as shown in Fig. 4.

**Fig. 5:**
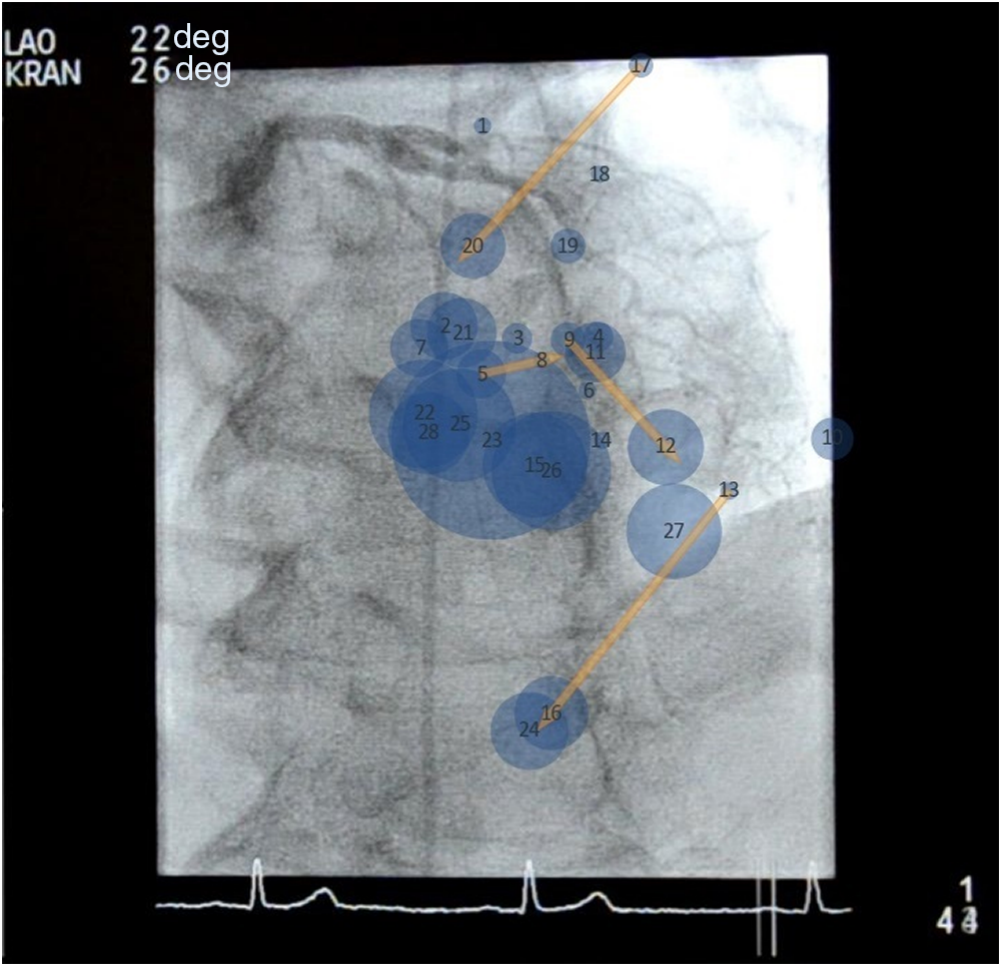
A scan path of the expert’s gaze in a single projection. Shown is a coronary angiogram in LAO cranial projection with operator’s gaze points centered on segment 7 stenosis (AHA/ACC nomenclature). The line shows fast movement between these fixations.

The data were displayed as a scan path, projecting the gaze locations and dwell times directly on the representative frame of the individual cine-series or a target DSA frame. The size of the circle around the gaze location is proportional to the dwell time (Fig. 5).

### 2.3 Statistical Analysis

Based on the assumption that the difficulty of a given procedure is reflected by its length of time, amount of radiation, and contrast agents needed to generate images, the procedure derived data included: fluoroscopy time, air kerma, surface dose product, and amount of the contrast agent, all of which are surrogates for the complexity of the procedure. All variables were registered automatically by the x-ray imaging system individually in each patient. Means and standard deviations were calculated for CA and PCIs.

The eye-tracking data were analyzed to calculate percent time spent on-screen and off-screen for all procedures. These measures were of interest because they reflect how much time the operator spent looking at the images (on-screen) versus conducting other activities (off-screen), such as selecting instrumentation, instructing personnel, and establishing rapport with the patient. As this was a pilot study, qualitative assessments were also made to visually compare the patterns generated in the three cases viewed by both the EO and NO.

## 3 Results

All interventional procedures were performed without any complications; no study-related side effects occurred. Procedure data are summarized in Table 1.

**Table 1:** Procedure data.

Case number	Age (year)	Procedure/diagnosis	Target vessel	Fluoro time plus cine time (min)	Air kerma (mGy)	Surface dose product ($\mathrm{dGy}/{\mathrm{cm}}^{2}$)	Contrast agent (ml)
1	49	PCI/NSTEMI	LAD/Rd	7.1	677	498	150
2	83	PCI/ ACS	LAD/LCx	8.9	515	393	190
3	75	PCI/CAD	RCA	5.1	732	499	120
4	56	PCI/CAD	LAD	7.8	2547	1659	300
5	70	PTA/PAD	SFA	3.2	256	1680	70
6	61	Coro/CAD	—	1.4	122	1068	60
7	76	Coro/CAD	—	0.9	171	1337	60
8	66	AP/CAD	LCx	4.2	449	2695	90
9	73	Coro/CAD	—	1.2	232	2472	50
10	64	Coro/CAD	—	1.6	490	3563	40
11	77	CAS/IBD	ACI left	31.6	1542	15241	190
12	56	Coro/CAD	—	1.1	112	980	50
13	77	Coro/CAD	—	1.1	185	1848	50
14	70	PCI/CAD	LAD	9.2	—	14035	270

Note: Coro, CA; PCI, percutaneous coronary angiography; CAD, coronary artery disease; IBD, ischemic brain disease; PTA, percutaneous transluminal angioplasty; ACI, carotid internal artery; ACS, acute coronary syndrome; NSTEMI, non-ST-segment elevation myocardial infarction; SFA, superficial femoral artery; LCx, left circumflex coronary artery; LAD, left anterior descending coronary artery; and RCA, right coronary artery.

Case number 2 was a technically demanding left anterior descending (LAD) and left circumflex (LCx) PCI in a patient with complex and diffuse coronary artery disease. Case number 4 was complex due to the diffuse character of the LAD lesions, high vulnerability of the vessels, and propensity to dissections requiring multiple stenting. Case number 11 was a technically demanding CAS intervention in a patient with a difficult access to the target site due to a Myla type III aortic arch and severely elongated left common carotid artery. Case number 14 was technically complex due to the presence of multiple hemodynamically relevant coronary artery plaques of the LAD. The other cases were considered routine by the EO.

**Fig. 6:**
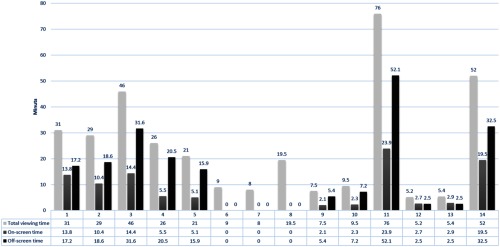
Bar diagram: summary of the on-screen time and off-screen time for all cases.

Quantitative analysis of gaze duration showed that the total viewing time of the EO was divided unevenly between on-screen and off-screen. The percentage of on-screen time per procedure varied between 21% and 70% of the total recorded procedural time. On-screen time was between 24% and 54% (mean=39.5%±13.6%) and 21% and 70% (mean=41.5%±18.3%) in CA and CA plus PCI procedures, respectively. An analysis of variance using percent time as the dependent variable and on- versus off-screen and performance or absence of PCI in addition to coronary angiography as the independent variables revealed that significantly more time was spent off-screen than on (F=10.17 and p=0.0051). There was no significant difference as a function of PCI versus no PCI. There was no significant difference in total viewing time (t=0.936 and p=0.3676) as a function of PCI or not PCI. The most complex coronary procedure (case number 2) was associated with the largest proportion of on-screen time. Table 2 and Fig. 6 summarize the viewing time results.

**Table 2:** Total viewing time on- and off-screen. For technical reasons, the on-/off-screen time in cases 6 to 8 could not be calculated.

Case number	Age (year)	Total viewing time (min)	On-screen time (min/% total)	Off-screen time (min/% total)
1	49	31	13.8/44%	17.2/56%
2	83	29	10.4/36%	18.6/64%
3	75	46	14.4/31%	31.6/69%
4	56	26	5.5/21%	20.5/79%
5	70	21	5.1/24%	15.9/76%
6	61	9	—	—
7	76	8	—	—
8	66	19.5	—	—
9	73	7.5	2.1/28%	5.4/72%
10	64	9.5	2.3/24%	7.2/76%
11	77	76	23.9/31%	52.1/69%
12	56	5.2	2.7/52%	2.5/48%
13	77	5.4	2.9/54%	2.5/46%
14	70	52	19.5/38%	32.5/62%

**Fig. 7:**
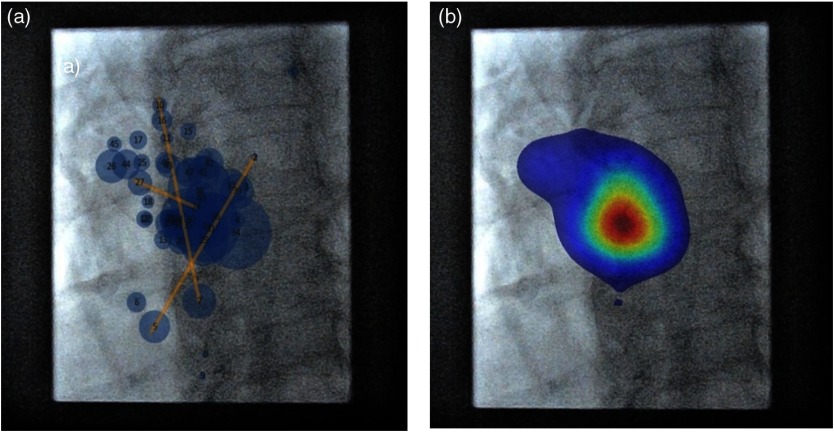
LAD intervention and gaze locations. Shown is a representative case gaze of an EO while performing PCI (case number 4). In cranial LAO projection, the LAD segment 7 lesion is projected approximately at the center of the screen. (a) The scan path, size of the circle corresponds to the dwell-time. (b) The heatmap of the scan path, the red color representing the most visited location on the x-ray image and the blue representing the least visited location.

The gaze location data revealed that, in the standard views, the EO typically looked at the center of the images at approximately eye level. When suspicious findings were identified, the EO redirected his gaze to explore these areas in detail. To explore suspicious findings in detail, additional projections were acquired. In these additional projections, the EO positioned the suspicious findings approximately in the center of the monitor fordirect viewing. Intermittent gazes directed at nontarget sites were also recorded (Fig. 7).

**Fig. 8:**
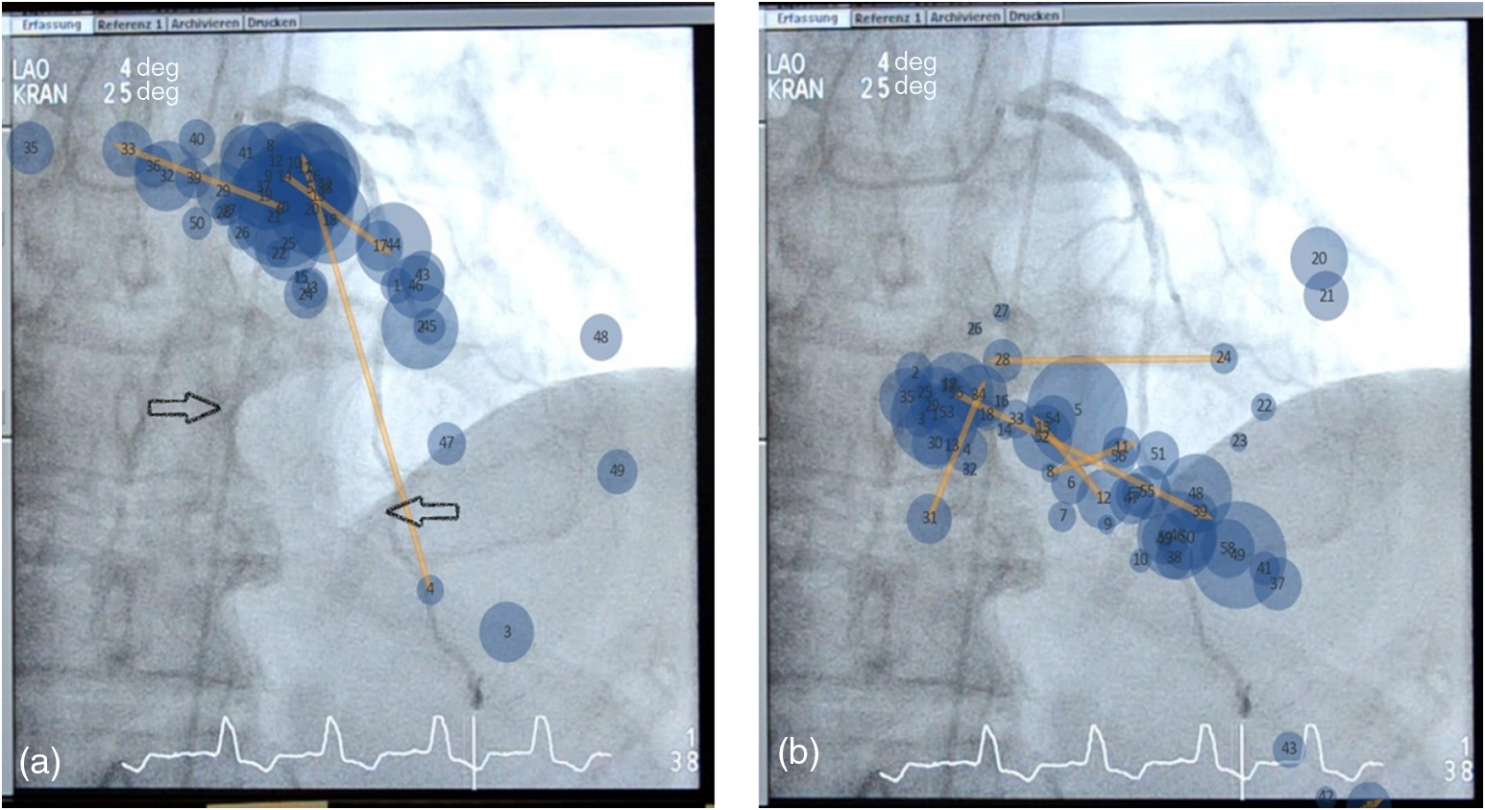
The scan paths of the EO and NOs. Shown is a representative frame of cranial 4-deg LAO 15-deg CA projections (case number 14). While the EO focuses his gazes on the area of the missing LCx to discern traces of the vessel (a, arrows), the NO continues to focus his gazes on the LAD lesions already sufficiently documented in earlier cine-image series (b).

In the three cases that both the EO and NO viewed (cases number 12-14), qualitative differences were noted. The NO typically fixated along the course of the coronary arteries, likely in an attempt to evaluate each American Heart Association/American College of Cardiology (AHA/ACC) coronary segment in a linear fashion. In case number 14, a patient with three vessel disease and a chronically occluded LCx, the EO looked at the LCA to assess the presence of a stump and the status of the collateral vessels. In all of these projections, the NO repeatedly focused on the diseased LAD and missed the absence of LCx. Figure 8 shows the gaze patterns of the EO and NO on a representative case.

## 4 Discussion

Understanding the EOs’ strategy to read a large number of dynamic and static x-ray images on-line and in real time correctly and efficiently, and frequently under time constraints in emergency settings, represents the first step in decoding the complex perceptual and cognitive processes required to perform CBCVI.^5^ To the best of our knowledge, this report represents the first attempt to analyze this first step in the complex CBCVI process in a real-life context.

The methodology developed in this study allows eye tracking of operators in real-life CBCVI cases. The methodology is safe. All procedures were performed according to the established SOPs, exposing the patient to no additional procedural risk. Since all procedures were performed in real life and in a standard manner, no artificial or confounding variables other than those attending any real-life intervention were introduced.

The results show that, in CBCVI performed by an EO, on average more than 50% of total procedural time was spent off-screen. During this off-screen time, the EO conducted activities not related to XRIP, such as observing the electrocardiogram and blood pressure monitor, selecting and preparing the instrumentation, communicating with the staff, and maintaining rapport with the patient. Switches between looking on- and off-screen require visual adjustments to compensate for substantial differences in luminosity between the screen and the surroundings. Although, we did not measure this directly (for example, by measuring pupil diameter), future studies could do so. On-screen time corresponds to XRIP. The EO reviewed the images by replaying the cine-loops in real-time or slow-motion, scrolling through the loops frame by frame back and forth, or studying individual images.

In all cases, the EO examined the images with the main area of interest positioned in the center of the monitor. If no abnormalities were noted, the EO did not divert attention (gaze) from the monitor center. If abnormalities were suspected in standard projections, the EO directed his gaze to the suspicious area. In all subsequent projections, the suspicious area was the new center, allowing the EO to maintain his central focus. This finding underscores the importance of properly centering the images to allow the operator to maintain a single point of attention (focus), thus likely minimizing the cognitive effort needed to keep readjusting gaze between series. These data confirm earlier findings where imaging and interventional nonCBCVI procedures were assessed with eye tracking.^6^^–^^10^

Interestingly, in the three studies performed by the EO and observed by the NO, the viewing strategies differed. The EO viewed the images in standard projections maintaining gaze at the center of the monitor and shifting only if suspicious findings were detected. In contrast, the NO employed an exhaustive approach painstakingly examining the standard and additional images by moving his gaze systematically in segment-by-segment fashion. In one case, an occluded coronary artery (LCx) was missed by the NO who focused on the interrogation of the multiple LAD lesions.

The amount of on- and off-screen time varied among the procedures. There was only a minor increase in on-screen time in CA with PCI versus CA alone (41.5%±18.3% versus 39.5%±13.6%). In more complex CBCVIs, the on-screen time was longer.

The study shows that the developed methodology can be employed safely and efficiently in real-life CBCVI settings without introducing confounding variables associated with the artificial conditions of experimental models and computer simulations. Based on this initial experience, we confirmed that using portable eye-tracking technology to analyze XRIP strategies of operators in real-life CBCVI is feasible. Analysis of strategies employed by EOs could, in the future, help develop methods to educate and train less experienced operators.^18^

### 4.1 Study Limitations

This study represents an early stage of inquiry into the complex visual processing required to develop expertise in CBCVI. Expertise in this case requires correct image interpretation and translation of image data into interventional strategies and tactics, including selection of the most appropriate case-specific instrumentation and manual conduct of the interventional actions. Extending this method to other techniques, such as functional magnetic resonance imaging, will be needed to unravel the intricacies of CBCVI decision-making and motor skills. Furthermore, more advanced analytical tools still need to be developed to expedite the image data matching process and to pinpoint more specifically the features of interest viewed by the operators. In this pilot study, only one EO and one NO participated (and the novice not on all cases). To be a useful and practical tool in teaching and training CBCVI, larger studies are needed to properly characterize, explicate, and eventually transfer the EOs’ XRIP strategies into CBCVI curricula.

## 5 Summary

This study demonstrates for the first time the potential to gain insight into the cognitive and perceptual strategies associated with CBCVI. Based on this proof of the principle of eye-tracking data acquisition in real-life scenarios, we were able to gain early insights into the x-ray image viewing strategies of an expert interventionist and NO. Using the established protocol, the conduct of more systematic studies with multiple operators with different levels of expertise is now feasible. Data hold the potential to optimize viewing strategies and to instruct learners. In addition, insights into the chain of the complex cognitive functions associated with judgments, decision–making, and action taking in CBCVI appear now to be feasible.
